# Abortive and Propagating Intracellular Calcium Waves: Analysis from a Hybrid Model

**DOI:** 10.1371/journal.pone.0115187

**Published:** 2015-01-20

**Authors:** Nara Guisoni, Paola Ferrero, Carla Layana, Luis Diambra

**Affiliations:** 1 Instituto de Física de Líquidos y Sistemas Biológicos (IFLYSIB), Universidad Nacional de La Plata, CONICET CCT-La Plata; Calle 59–789 (1900) La Plata, Argentina; 2 Centro de Investigaciones Cardiovasculares, Facultad de Ciencias Médicas; 60 y 120 (1900) La Plata, Argentina; 3 Centro Regional de Estudios Genómicos (CREG), Universidad Nacional de La Plata; Blvd 120 N 1461 (1900) La Plata, Argentina; University of Western Sydney, AUSTRALIA

## Abstract

The functional properties of inositol(1,4,5)-triphosphate (IP_3_) receptors allow a variety of intracellular Ca^2+^ phenomena. In this way, global phenomena, such as propagating and abortive Ca^2+^ waves, as well as local events such as puffs, have been observed. Several experimental studies suggest that many features of global phenomena (e.g., frequency, amplitude, speed wave) depend on the interplay of biophysical processes such as diffusion, buffering, efflux and influx rates, which in turn depend on parameters such as buffer concentration, Ca^2+^ pump density, cytosolic IP_3_ level, and intercluster distance. Besides, it is known that cells are able to modify some of these parameters in order to regulate the Ca^2+^ signaling. By using a hybrid model, we analyzed different features of the hierarchy of calcium events as a function of two relevant parameters for the calcium signaling, the intercluster distance and the pump strength or intensity. In the space spanned by these two parameters, we found two modes of calcium dynamics, one dominated by abortive calcium waves and the other by propagating waves. Smaller distances between the release sites promote propagating calcium waves, while the increase of the efflux rate makes the transition from propagating to abortive waves occur at lower values of intercluster distance. We determined the frontier between these two modes, in the parameter space defined by the intercluster distance and the pump strength. Furthermore, we found that the velocity of simulated calcium waves accomplishes Luther’s law, and that an effective rate constant for autocatalytic calcium production decays linearly with both the intercluster distance and the pump strength.

## Introduction

Cytosolic-free calcium (Ca^2+^) is a ubiquitous intracellular messenger for regulating a diverse range of cellular processes, such as gene transcription, muscle contraction, secretion, fertilization, and cell proliferation. In order to control this variety of functions, calcium is precisely regulated in space and time. In cells that are not electrically excitable, calcium is stored in the endoplasmic reticulum (ER). Changes in the intracellular Ca^2+^ concentration are due fundamentally to the exchange between the cytosol and the ER. In this way, part of the calcium stored in the ER can be released by a variety of channels that form a set of sensory and release mechanisms. In particular, the inositol(1,4,5)-triphophate receptor (IP_3_R) displays an autocatalytic amplification, since it is active when the IP_3_ and only one Ca^2+^ are bound to the receptor. This mechanism is called calcium-induced-calcium-release (CICR), since low Ca^2+^ levels in the cytosol favor channel opening. The receptor becomes inactive when a calcium ion is bound to the inhibitory binding site, rendering a highly nonlinear behavior. The cytosolic Ca^2+^ is removed by energy-dependent pumps, such as the sarco-endoplasmic reticulum ATPases (SERCAs), to be stored in the ER.

It has been observed that Ca^2+^ release channels are spatially organized in clusters [1]. Due to the CICR mechanism, the Ca^2+^ release by a channel increases the open probability of the neighboring channels, which conduces to the collective opening (and closing) of several Ca^2+^ channels in a cluster, an event named “puff”, as observed in early experiments [2]. Also, neighboring clusters can become functionally coupled by Ca^2+^ diffusion, and CICR supports the formation of intracellular Ca^2+^ waves. Calcium waves that travel long distances over the cell are called “propagating waves”, whereas those ones that vanish relatively close to the region of initiation are referred to “abortive waves”. The variability in properties such as amplitude, period and velocity of a propagating calcium wave generates a huge repertory in the signal transmission. In fact, Ca^2+^ is considered one of the most important second messengers and calcium waves can be understood as an encoding tool for cell signaling [3].

There are several experimental and theoretical studies about calcium waves. Jaffe [4] compiled data from 42 different systems that exhibit intracellular calcium waves in both activating eggs and in fully active cells. In all cases the waves are shown to travel from one pole of a cell to the other, or from the periphery towards the inside of the cell. The velocities of these waves are remarkably conserved, from 5 to 14 *μ*m/s in activating eggs, and from 15 to 40 *μ*m/s in other cells at room temperature. Marchant *et al*. have reported that the velocity of these waves increases with IP_3_ concentration [5]. Camacho and Lechleiter [6, 7] have investigated the key role of Ca^2+^ pump density by overexpressing two types of SERCA in *Xenopus* oocytes. Surprisingly, by increasing the Ca^2+^ pump density of both SERCA isoforms, they observed a decrease in the period and an increase in the amplitude of intracellular Ca^2+^ waves. It was also observed that the overexpression of the SERCA type that has a smaller pump capacity and a higher Ca^2+^ affinity increases the calcium wave velocity [8], whereas overexpression of the other SERCA isoform does not change the velocity substantially [6]. The dependence of Ca^2+^ pump density on the velocity of calcium waves was also analysed in theoretical studies by means of deterministic models [9, 10].

From the theoretical point of view, the effect of the spatial distribution of release sites on calcium waves was also studied [10–15]. By using deterministic models for calcium release, Dupont and Goldbeter [10] found that the velocity and the period of calcium waves drop significantly as the distance between release sites increases, in agreement with other theoretical results [12–15]. Diambra and Marchant [11] used a high-resolution model that incorporates the stochastically gating IP_3_R and showed that the velocity of a microwave front decreases as a function of the interchannel distance.

Experimentally, release pattern events composed of puffs, abortive and propagating waves were observed in *Xenopus* oocytes by increasing the level of the channel activator IP_3_ [5, 16]. Also, in *Xenopus* oocytes loaded with caged IP_3_, abortive waves have been observed when the IP_3_ level is just below the threshold for wave propagation [17]. Therefore, by increasing the IP_3_ level, it is possible to see a transition from limited propagation of abortive waves to stable propagating waves. Bugrim *et al*. [14] have considered a random distribution of release sites in a deterministic model for *Xenopus* oocytes to study calcium wave propagation. They located a transition line between abortive and propagating waves, so that lower IP_3_ concentration and high average intercluster distance favor abortive waves. Other authors have studied the universal properties of the transition between these different kinds of propagation phenomena, and found that it belongs to the directed percolation universality class [18–23].

Despite the existence of several experimental and theoretical studies about calcium dynamics, the relationship between several quantitative features and relevant control parameters of the system is still incomplete. Particularly, most previous mathematical models focused on the characterization of calcium waves are basically deterministic. However, the bindings of IP_3_ and Ca^2+^ to the regulatory sites of the receptor are stochastic events rendering the opening and closing of the channel a stochastic process. Also, the observation of localized stochastic Ca^2+^ puffs suggests that it is mandatory to take into account stochasticity for modeling local Ca^2+^ releases. In fact, abortive waves cannot be understood in terms of deterministic models, since in these models Ca^2+^ waves travel indefinitely. In the present work, we use a hybrid model of spatially distributed IP_3_R clusters that accounts for different calcium release events: puffs, abortive and propagating waves. We studied the effect of two relevant parameters for calcium propagation, the intercluster distance and the efflux rate through SERCA pumps. Calcium release events are shown to exist only for a certain range of these parameters. We found that abortive and propagating waves coexist, and we identified a frontier between a region of the parameter space dominated by abortive calcium waves and another where propagating waves are prevalent. Also, we determined the velocity of propagating calcium waves and studied an effective rate constant for autocatalytic calcium production. A statistical analysis of number, duration and amount of calcium release in the different events was made.

## Methods

The hybrid model considered here is defined by a partial differential equation for the Ca^2+^ dynamics in the cytoplasm and a Markov-stochastic kinetic model for the individual IP_3_R channels. Our model considers four contributions of the Ca^2+^ dynamics: diffusion of cytosolic Ca^2+^, calcium influx from the ER through the IP_3_R channels, calcium efflux pumped by the SERCAs into the ER, and calcium influx leakaged through the ER membrane to the cytosol. The number of actual open channels is determined by stochastic simulations of individual IP_3_R channels. On the other hand, the transition rates between the different states of a given IP_3_R depend on the cytosolic Ca^2+^ concentration at the channel position, which is in turn determined by numerical integration of the partial differential equation for the Ca^2+^ dynamics.

### Calcium dynamics model

We consider the dynamic of Ca^2+^ in the cytoplasm as governed by the equation

$$\frac{d\left[{\mathrm{Ca}}^{2+}\right]}{dt}=D\nabla^{2}\left[{\mathrm{Ca}}^{2+}\right]+J_{channel}+J_{leak}-J_{pump},$$ (1)

where [Ca^2+^] is the Ca^2+^ concentration in the cytosol, which can diffuse with diffusion constant D. *J_channel_* is the calcium flux from the ER to the cytoplasm through the IP_3_R channels, *J_leak_* is the calcium flux leakaged from the ER to the cytoplasm, and *J_pump_* is the calcium flux pumped by the SERCAs from the cytoplasm into the ER. The expressions for the fluxes are given by

$$\begin{array}{ccc} J_{channel} & = & v_{r}\gamma_{1}N_{open}\left({\left[{\mathrm{Ca}}^{2+}\right]}_{\mathrm{ER}}-\left[{\mathrm{Ca}}^{2+}\right]\right)\\ J_{leak} & = & v_{r}\gamma_{0}\left({\left[{\mathrm{Ca}}^{2+}\right]}_{\mathrm{ER}}-\left[{\mathrm{Ca}}^{2+}\right]\right)\\ J_{pump} & = & \hat{p}\frac{{\left[{\mathrm{Ca}}^{2+}\right]}^{2}}{{\left[{\mathrm{Ca}}^{2+}\right]}^{2}+{\hat{q}}^{2}}, \end{array}$$ (2)

where *v_r_* is the ratio of the ER volume to the cytoplasm volume, [Ca^2+^]_ER_ is the Ca^2+^ concentration in the ER, *γ*_1_ is the maximal Ca^2+^ fluxes per channel, *N_open_* is a random variable that represents the number of open channels, and *γ*_0_ is the basal permeability of the ER membrane in the absence of IP_3_. The parameter $\hat{p}$, named pump flux capacity, regulates the Ca^2+^ efflux from the cytoplasm into the ER. Note that we do not make a distinction between the efficiency of the pump and the SERCA density; both features are represented by the parameter $\hat{p}$. As usual, the pump action is modeled by a sigmoidal curve with Hill coefficient equal to 2 and activation threshold $\hat{q}$ [9]. We neglect Ca^2+^ exchange with the extracellular medium since it is much smaller than the Ca^2+^ flux across the ER membrane [24]. For this reason, Eqs. 1 and 2 can be simplified by using the volume average intracellular calcium concentration [Ca^2+^]_ave_ = ([Ca^2+^] + *v_r_* [Ca^2+^]_ER_)/(1 + *v_r_*). The [Ca^2+^]_ave_ is a control parameter whose value we can choose, but it is not a dynamical variable. We then define C = [Ca^2+^]/[Ca^2+^]_ave_ and rewrite Eqs. 1 and 2 in the form

$$\begin{array}{ccc} \frac{dC}{dt} & = & D\nabla^{2}C+\left(1+v_{r}\right)\left(\gamma_{1}N_{open}+\gamma_{0}\right)\left(1-C\right)\\ & & -p\frac{C^{2}}{C^{2}+q^{2}}, \end{array}$$ (3)

where $p=\hat{p}/{\left[{\text{Ca}}^{2+}\right]}_{\text{ave}}$ and $q=\hat{q}/{\left[{\text{Ca}}^{2+}\right]}_{\text{ave}}$. The finite-difference method is used to solve the partial differential Eqs. 3, with Δ*x* = 0.18 *μ*m and Δ*t* = 0.0005 s. The parameters of the model are given in Table 1. We consider different values of the pump strength *p* in the range 1.2 and 3.0 *μ*M s^-1^.

**Table 1:** Parameter values for the model. Other parameters are given in the figure captions.

Dynamic parameters		Kinetic parameters	
name	value	name	value
[IP_3_]	0.5 *μ*M	[Ca^2+^]_ave_	1.6 *μ*M
*v_r_*	0.185	*k*_1_	24.0 (*μ*M × s)^-1^
*γ*_0_	0.02 s^-1^	*k*_-1_	32.0 s^-1^
*γ*_1_	9.0 s^-1^	*k*_2_	30.0 (*μ*M × s)^-1^
*D*	25 *μ*m^2^ s^-1^	*k*_-2_	0.82 s^-1^
$\hat{q}$	0.01 *μ*M	*k*_3_	3.6 (*μ*M × s)^-1^
$\hat{p}$	[1.2 – 3.0] *μ*M s^-1^	*k*_-3_	0.064 s^-1^

### Kinetic model of IP_3_-receptor and calcium channel

**Figure 1:**
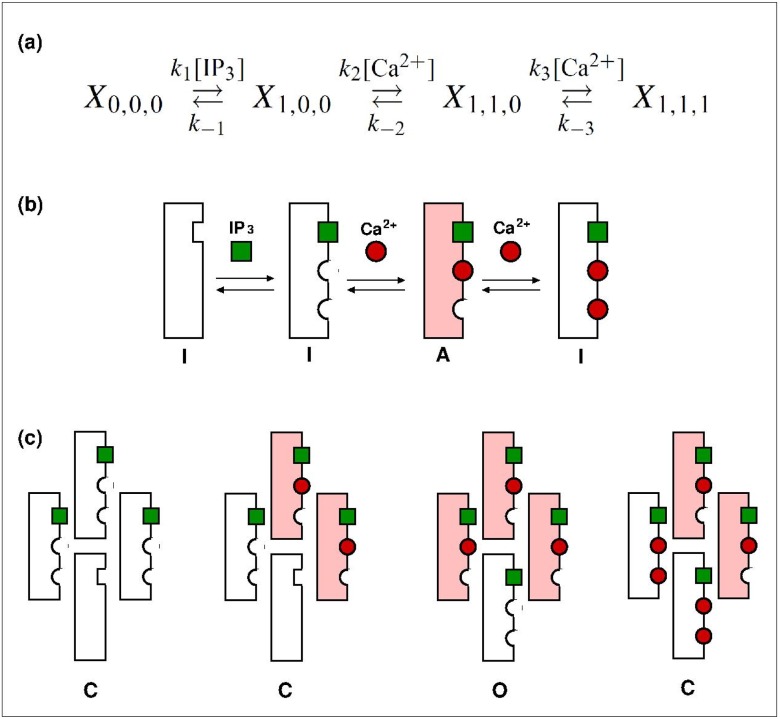
Schematic representation of the stochastic model. (a) Scheme of the four-state model for the IP_3_R and the allowed transitions. The states are represented by *X_i, j, k_*, where the subindex *i* stands for the IP_3_ binding site, *j* for the activator Ca^2+^ binding site, and *k* for the inhibitory Ca^2+^ binding site. For each binding site, a subindex value equal to 1 represents an occupied site, otherwise the site is unoccupied. *k_i_*, *i* = ±1, ±2, ±3, is the rate constant of each state transition. (b) Representation of the states and allowed transitions of the IP_3_R, which can be inactive (I) (in states X_0,0,0_, X_1,0,0_ and X_1,1,1_) or active (A) (state X_1,1,0_). (c) Example of some configurations of the four-subunit channel (we show just 4 of the 256 possible configurations for the channel). The channel is open (indicated by O) only when 3 or 4 subunits are in the active state, otherwise it is closed (C).

The IP_3_ receptor is modeled by a stochastic version of the Othmer and Tang (OT) model [25, 26]. This kinetic model considers three regulatory sites for a receptor: one for IP_3_, one activating site and one inhibiting site for Ca^2+^. Ca^2+^ binds to the activating site on the receptor only after IP_3_ has bound, and the binding of calcium to the inhibitory site occurs only after calcium is bound to the activating site. This sequential binding leads to four possible states for a receptor as shown in Figs. 1-A and 1-B. The receptor is active only when there is one IP_3_ bound and one Ca^2+^ bound to the activating site (corresponding to X_1,1,0_).

In the original OT model the channel is a monomer, i.e., it is represented by only one IP_3_-receptor subunit described above [25–27]. In the present work we take into account the experimental fact that the calcium channels are tetrameric structures composed of four IP_3_-receptor subunits [28]. Therefore, we represent each channel as composed of four identical and independent subunits (i.e., the binding or unbinding of IP_3_ and Ca^2+^ to the regulatory sites of a subunit is not affected by the state of the other subunits that compose the channel), as represented in Fig. 1-C. Also, the channel is considered open only when three or four subunits are active [29, 30]. The parameters of the kinetic model are given in Table 1.

### Simulations and Analysis

**Figure 2:**
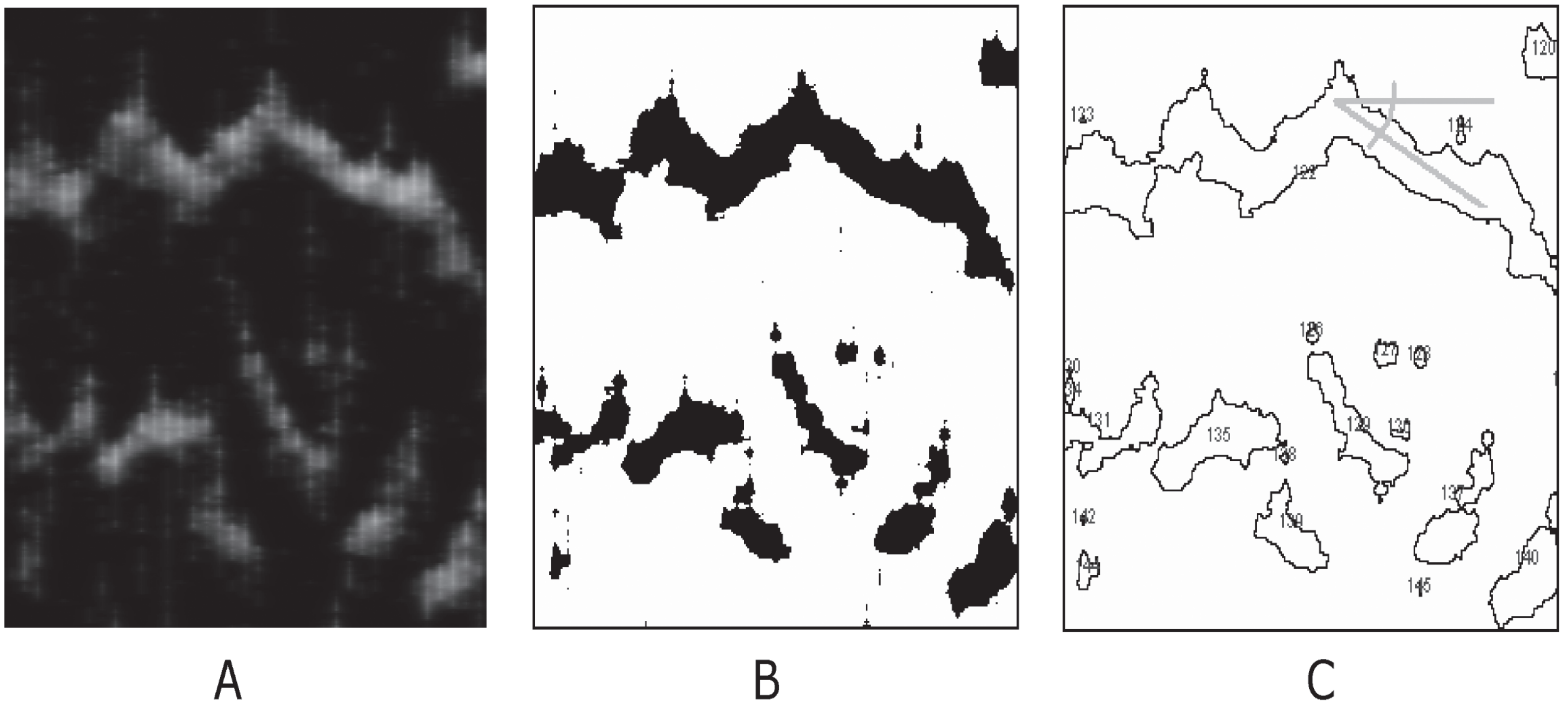
Analysis of spatial-temporal profiles. A: Calcium density plot showing a typical outcome of the model simulation (horizontal axes: space, vertical axes: time). B: Thresholded image, black spots are regions where the Ca^+2^ level exceeds 0.3 *μ*M. C: All spots shown in B are counted and characterized (intensity, size, duration and velocity, i.e., angle *α*).

We assume that the Ca^2+^ channels are spatially organized into clusters of size *N* = 64. These channels evolve following the Markovian dynamics described above. The channel clusters are disposed in a one-dimensional array, with a fixed intercluster distance *d*. This geometry is useful to study different events of calcium release, as can be seen in Fig. 2-A that shows fragments of the typical outcome of the simulations, where the [Ca^2+^] along the one-dimensional space (horizontal axes) is represented in a gray scale.

In order to study the role of the intercluster distance *d* and the pump strength *p* in calcium dynamics, we perform 80 simulations varying these parameters and keeping the remaining one constant. The value of *d*, which varies between 1.6 and 3.7 *μ*m [5, 31], is controlled by using a variable number of clusters, which are uniformly distributed along a segment of 113 *μ*m (equivalent to 640 pixels on the output images). For all simulations we use periodical boundary conditions, and a small trigger signal in the middle of *x*-axes, as initial condition. For each simulation condition, determined by *d* and *p* parameter values, we perform 5 seconds (equivalent to 10000 time steps) of the calcium dynamics simulation and store the resulting temporal [Ca^2+^] profile with eight-bit resolution for subsequent analysis with ImageJ program [32] (see Fig. 2).

Our analysis of the temporal [Ca^2+^] profiles consists in the segmentation of the calcium release events from the images by using customized ImageJ scripts. These images are binarized with a threshold of 0.3 *μ*M for Ca^+2^ level (see Fig. 2-B). Then, by using the particle analyzer function of ImageJ, we characterize all detected calcium release events, and determine their duration, mean [Ca^2+^], and propagation distance. The recorded events are classified, following a size hierarchy, in: puffs, abortive and propagating waves. In this sense, we define two objective criteria in order to obtain the above classification. One criterion is based on the spatial distance spanned by the Ca^+2^ event. The typical distance between clusters is around 2.5 *μ*m, since the intercluster distance used here ranges between 1.6–3.7 *μ*m. Thus, a release event that propagates through a distance smaller than 5 *μ*m (twice the typical distance between clusters) is considered a puff. Abortive waves are events that propagate through distances between 5 and 25 *μ*m, while propagating waves spread over distances above 25 *μ*m. Besides the above spatial criterion, based on traveled distance, we also establish an alternative criterion in terms of the size of the event. In this sense, we define the total amount of calcium associated with each event, Ca_*T*_. In our simulation this quantity is estimated by multiplying the average calcium of the spot event by its area in pixels, in arbitrary units (a.u.). Again we classify the hierarchy of the events by setting thresholds on the amount of Ca_*T*_ in the event: puffs are events with total calcium of less than 200 a.u. If the Ca_*T*_ is between 200 a.u. and 1800 a.u. the event corresponds to an abortive wave, while if the Ca_*T*_ is above 1800 a.u. the event is defined as a propagating wave.

We also estimate the velocity of propagating waves by measuring the angle *α*, between the direction in which the calcium waves propagate and the horizontal axes, as can be seen in Fig. 2-C. This measurement is a computer-assisted manual procedure and is performed only for propagating waves. For each simulation output we calculate the propagation velocity of a significant number of waves, by considering that the wave velocity is given by *v_w_* = 0.18/ [tan(*α*)0.0375] *μ*m/s, in the same manner as in [5]. Then, we average the velocity over the recorded events in each simulation, which is considered as the typical velocity of the calcium wave propagation in such condition.

## Results

We observed that the events of Ca^+2^ release are limited to a region of the parameter space spanned by *d* and *p*. In this way, when the distance between clustered IP_3_Rs is shorter than 1.6 *μ*m and the intensity of calcium uptake by SERCA is smaller than 1.2 *μ*M/*s*, the high availability of Ca^+2^ observed in the cytosol prevents the identification of discrete Ca^+2^ release events. For distances longer than 3.7 *μ*m and pump strength greater than 3.0 *μ*M/s we observed a very small number of isolated events and the statistic is extremely poor. For this reason, we consider *d* ranging from 1.6 and 3.7 *μ*m, and *p* between 1.2 and 3.0 *μ*M/s.

**Figure 3:**
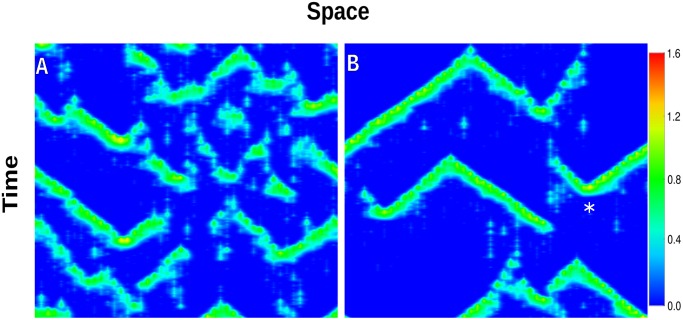
Typical outcomes of the model simulation. Calcium density in fragments of space (horizontal) × time (vertical) for *d* = 2.5 *μ*m. Whereas for *p* = 1.8 *μ*M/s different kinds of release events coexist (A), for *p* = 1.6 *μ*M/s global release events are prevalent (B). Two fronts annihilate each other upon collision (white star in B).

Fig. 3 shows the results of two simulations obtained with the hybrid model described above for an intercluster distance *d* = 2.5 *μ*m and two different values of *p*. From these profiles we can distinguish the presence of different kinds of release events: for a small value of *p* a variety of events, both local and global, can be observed (Fig. 3-A). When the pump strength is increased, the dynamics of calcium is dominated by global release events, i.e., events that are able to propagate in a sustained way both in time and in space (Fig. 3-B). Note that two fronts annihilate each other upon collision (white star in Fig. 3-B), as expected for excitable systems [10] and in agreement with experimental observations in oocytes [16, 33].

Since the number of detected puffs is in general one or two orders of magnitudes greater than the number of propagating or abortive waves (independently of the values of *d* and *p*), we compute the total number of waves and their durations, in order to obtain a more quantitative understanding of the events of calcium release presented by our model. We note that for the range of parameters studied here, abortive and propagating waves coexist in the same simulation, in agreement with experimental results [2].

**Figure 4:**
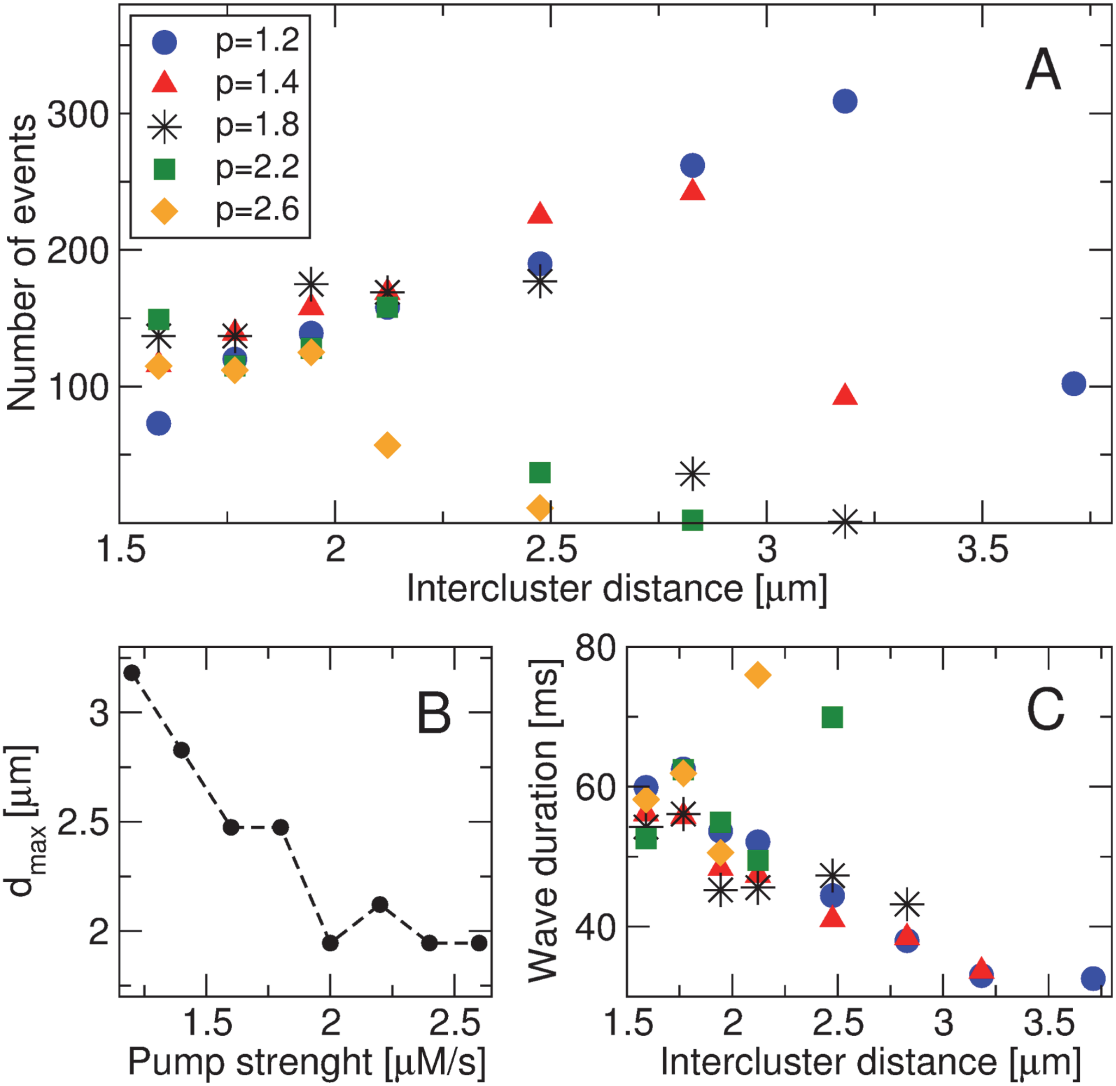
Statistical analysis of Ca^2+^ waves. (A) Number of Ca^2+^ wave release events *vs*. intercluster distance for different values of pump strength *p*. (B) Value of intercluster distance corresponding to the maximum number of Ca^2+^ waves (*d*_*max*_) as a function of *p*. (C) Mean wave duration *vs*. intercluster distance for different values of pump strength *p*. Waves are classified according to the spatial criteria (more details in the text).

Fig. 4-A shows the total number of waves (both propagating and abortive) as a function of the intercluster distance *d*, for different values of the pump strength *p*. For *p* ≤ 2.6 *μ*M/*s* we found that the number of events presents a maximum for a certain value of intercluster distance, which we call *d*_*max*_. The initial increase in the number of waves with the intercluster distance indicates that an excess of Ca^+2^ for the formation of waves is compensated by the increase in *d*, until a certain optimal distance (*d*_*max*_), from which the number of events decreases. Also, the value of *d*_*max*_ decreases with increasing *p*, as can be seen in Fig. 4-B. The effect of the pump strength on the number of waves is similar that of intercluster distance, which also presents a maximum for fixed values of *d* (not shown). On the other hand, the intercluster distance *d* also affects the wave duration. In Fig. 4-C we can see that the effect of *d* depends strongly on the pump strength: for *d* ≥ 1.8 *μ*m and small values of *p* (*p* ≤ 1.4 *μ*M/*s*) the wave duration decreases monotonically with the intercluster distance, whereas for higher values of *p* this behavior is not monotonic with *d*.

**Figure 5:**
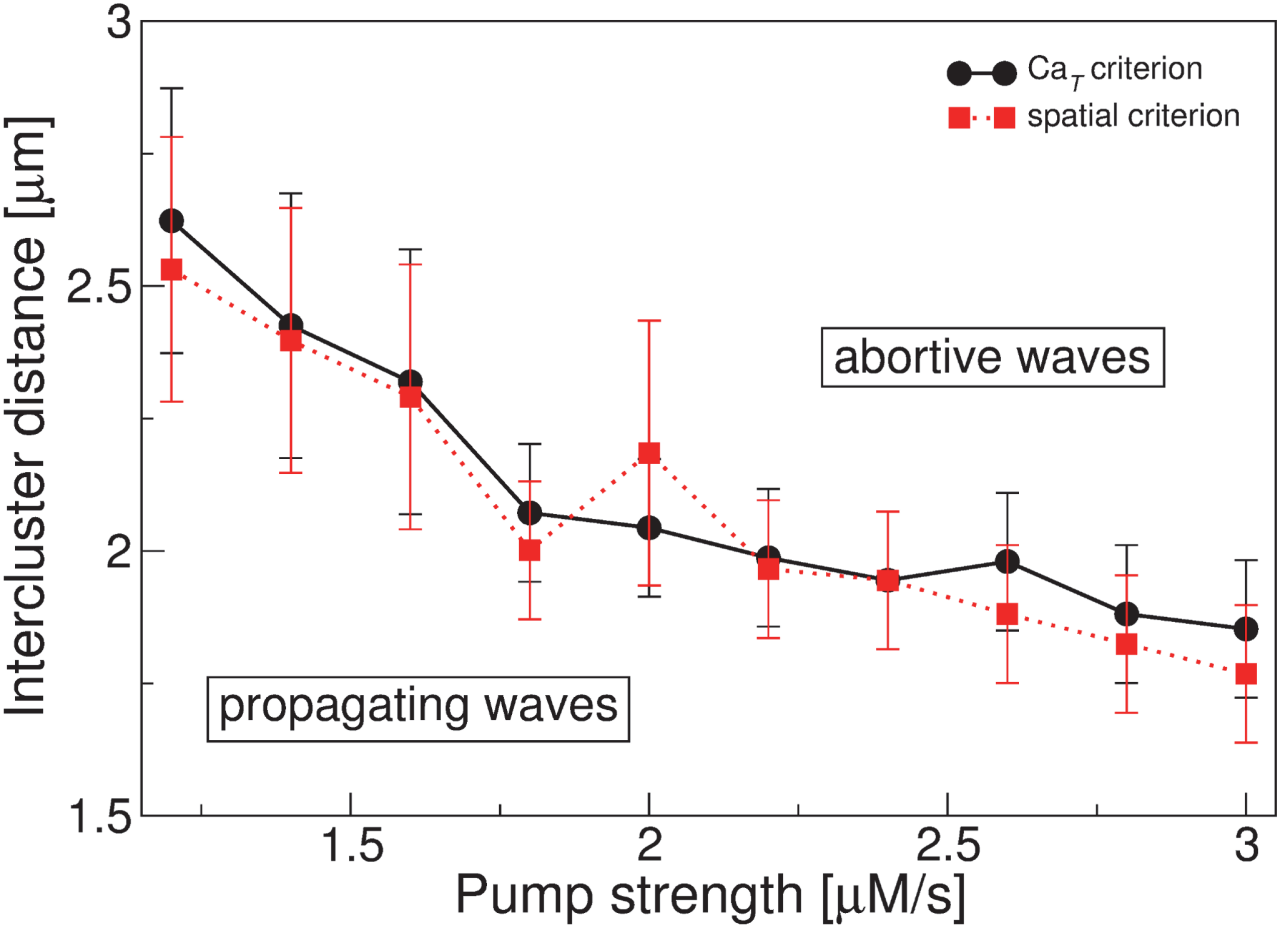
Phase diagram in the *d*–*p* space. The plot shows a region dominated by propagating waves (*n_w_* > *n_aw_*) and a region where the abortive waves are more abundant (*n_aw_* > *n*w**). Calcium release events are classified according to the spatial criterion (dashed line) and the calcium released criterion (solid line).

When comparing the number of propagating and abortive waves, we observed that for small values of *d*, the propagating waves are more abundant than the abortive waves, whereas for higher values of *d* the situation is inverted. A similar situation was found with respect to the pump strength, since an increase in *p* favors the presence of abortive waves. In this way, we can identify a region of the parameter space dominated by propagating waves (i.e., where the number of propagating waves, *n_w_*, is larger than that of abortive waves, *n_aw_*) and another region dominated by abortive waves (i.e., where *n_aw_* > *n_w_*). The points where *na_w_* = *n_w_* define a frontier between these regions, as can be seen in Fig. 5, where we used both the spatial criteria (dashed line) and the Ca_*T*_ criteria (solid line) in the characterization of the release events. Notice that the two different criteria used to characterize the type of calcium release events show good agreement to define the frontier. Also, we can see that by increasing *p*, the transition between the propagating region and the abortive region occurs at lower values of intercluster distance.

**Figure 6:**
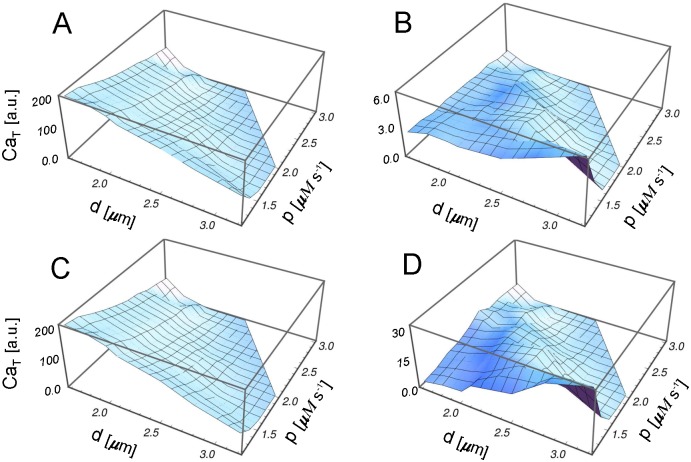
Ca_*T*_ for different release events. Total amount of calcium as a function of the pump strength *p* and the intercluster distance *d*. (A) Ca_*T*_ averaged over all events, (B) Ca_*T*_ averaged over puffs, (C) Ca_*T*_ averaged over propagating waves, and (D) Ca_*T*_ averaged over abortive waves. Vertical axes were scaled by a factor 10^6^. Note that the vertical scales are different.

**Figure 7:**
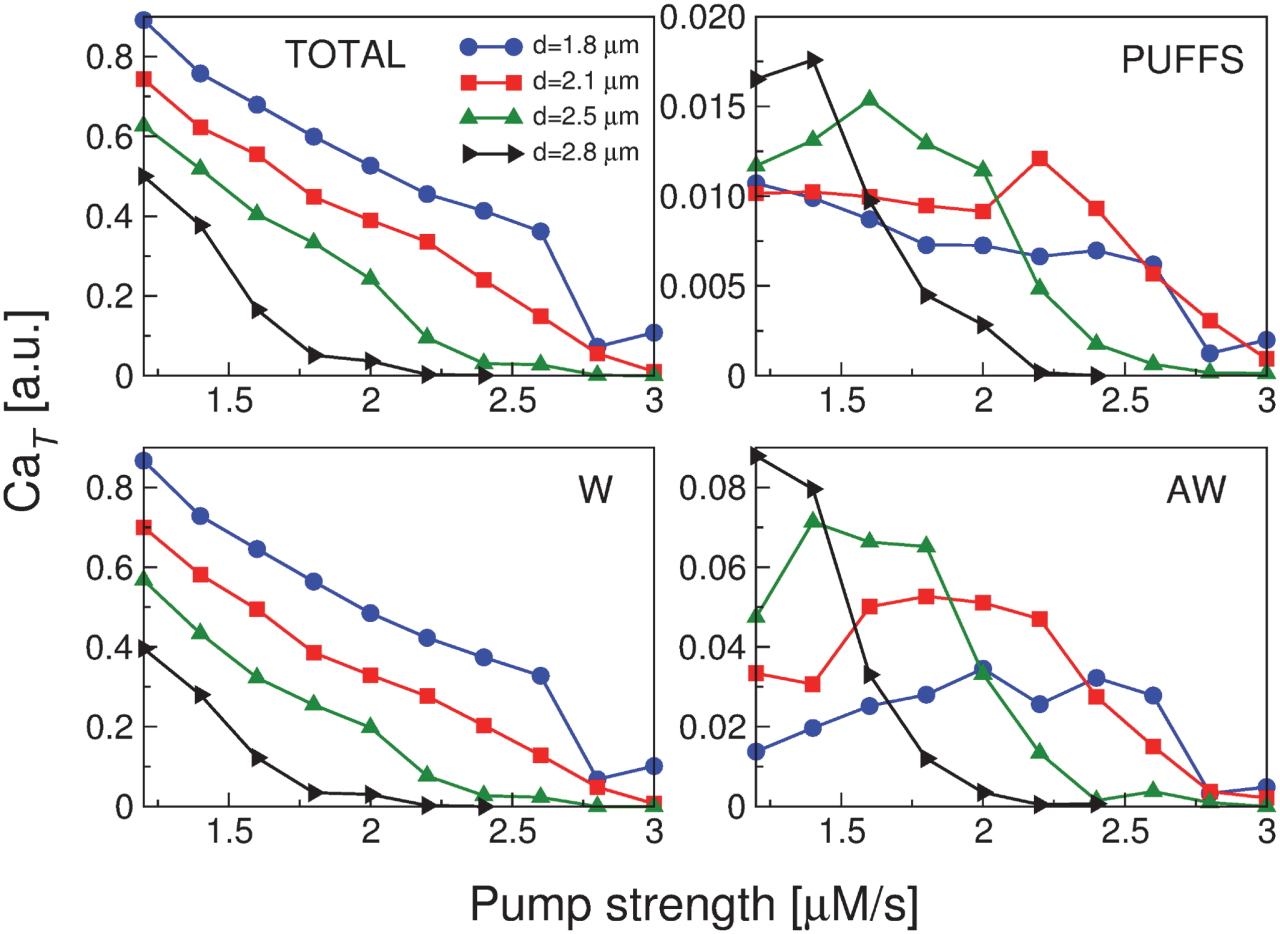
Ca_*T*_ *versus* the pump strength *p*. Total amount of calcium for different values of the intercluster distance *d*, as indicated. Ca_*T*_ averaged over all events (TOTAL), puffs (PUFFS), propagating waves (W) and abortive waves (AW). Vertical axes were scaled by a factor 10^6^. Note that the vertical scale for the figures is different.

In Fig. 6 we show the total amount of cytosolic calcium, Ca_*T*_, for the different events observed in our simulations as a function of *d* and *p*. From the comparison of Figs. 6-A and 6-C (corresponding to the total calcium in all events and the total calcium in propagating waves, respectively) we conclude that the propagating wave mode explains the bulk of Ca^2+^ released, almost independently of the parameter value. Also, whereas Figs. 6-A and 6-C are decreasing functions of *d* and *p*, the Ca_*T*_ related to both puffs and abortive waves (Figs. 6-B and 6-D, respectively) presents a region of maxima for intermediate values of *p* and *d*. It indicates that there is an optimal region in the parameter space *p*–*d* for the calcium release by these types of events. These results can also be seen in Fig. 7 where we show Ca_*T*_ for the different events as a function the pump strength *p* for several values of the intercluster distance *d*.

**Figure 8:**
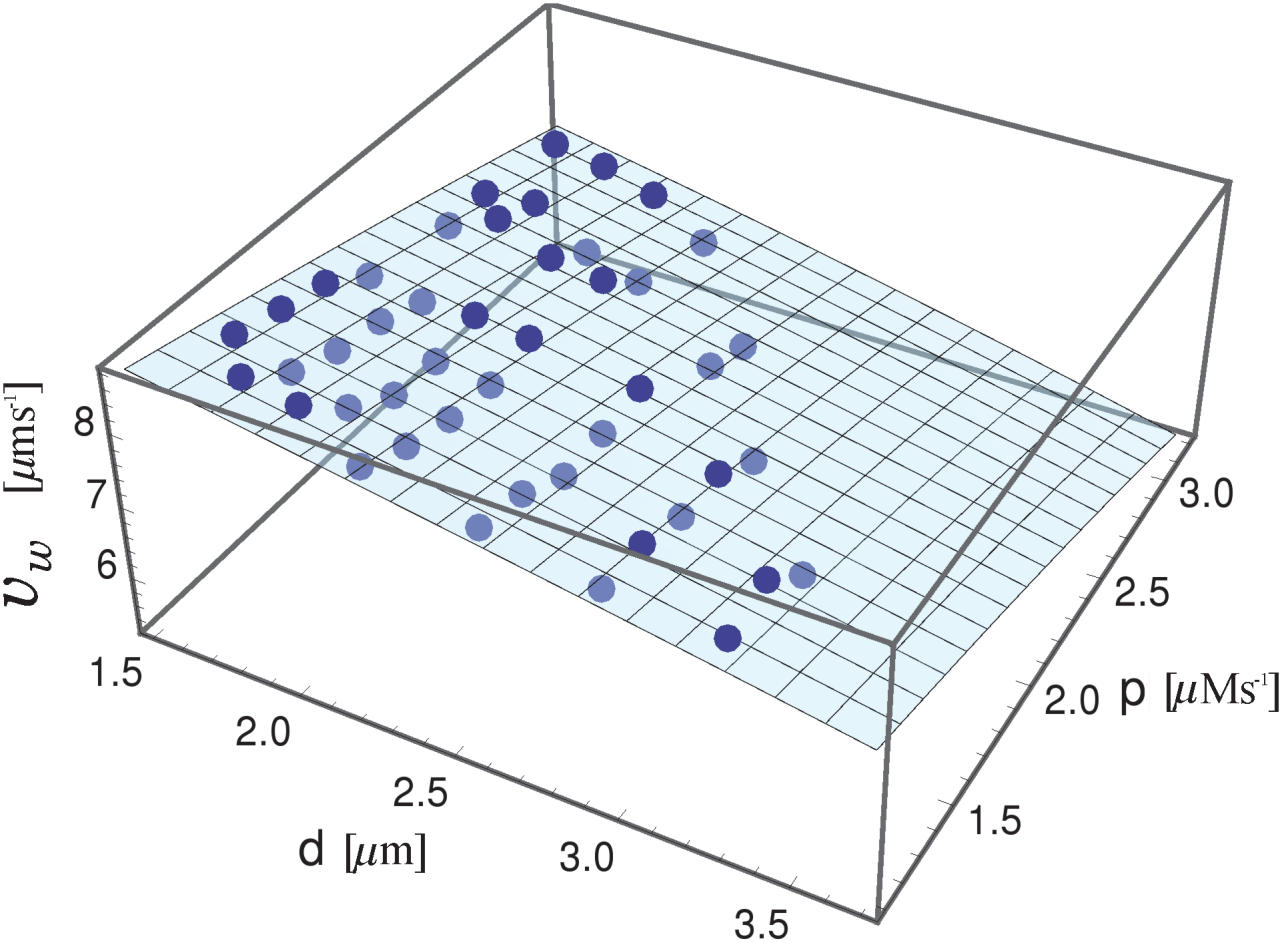
Velocity of calcium waves. Mean calcium wave velocity (*v_w_*) *vs*. the intercluster distance *d* and the pump strength *p*. Blue bullets: estimations obtained from the simulation results. The light-blue plane corresponds to the root square of the fitting: $v_{w}^{2}(d,p)=107.43-10.99d-12.19p$. The coefficient of determination of the data fitting proposed is *R* ^2^ = 0.83.

Finally, we studied the effect of the parameters *d* and *p* on the velocity of the wave front propagation. In Fig. 8 we show the average velocity of calcium waves *v_w_* (blue bullets) as a function of the intercluster distance *d*, and the pump strength *p*. We found that the velocities are in the range 6.6 *μ*m/s < *v_w_* < 9.0 *μ*m/s. Also, we observed that the velocity of wave propagation decreases with the parameters *d* and *p*, in agreement with other theoretical studies [10, 11, 14]. This behavior can be understood in terms of an effective first-order rate constant for the autocatalytic production of calcium. In this sense, we applied Luther’s law to the calcium waves obtained from our model [34, 35]. Followig Luther, we propose a relationship between the velocity of a calcium wave *v_w_*, the diffusion coefficient *D*, and the apparent first-order rate constant for autocatalytic calcium production *k*,

$$\begin{array}{c} v_{w}=a\sqrt{Dk}, \end{array}$$ (4)

where *a* is a dimensionless constant. Therefore, by adjusting a linear model to *k* obtained from the square calcium wave velocity $v_{w}^{2}$ (Fig. 8), we are able to predict that the effective first-order rate constant *k* decreases with both the intercluster distance *d* and the pump strength *p*, according to: *k*(*d, p*) = 4.30 - 0.44*d* - 0.49*p*, where we used *a* = 1 following [4, 13].

## Conclusion and Discussion

In the present work we propose a hybrid model of spatially distributed IP_3_R clusters by considering a partial differential equation for the Ca^2+^ dynamics in the cytoplasm and a Markov-stochastic kinetic model for the IP_3_R channels. The proposed model allows the simulation of global calcium release events such as abortive and propagating waves. Our main aim is to study how several features of the calcium events, such as: duration, wave propagation velocity, and number of events, are modulated by two relevant parameters for the calcium dynamics, the intercluster distance and the efflux rate through SERCA pumps.

The results of our simulations showed that waves are observed in a specific region of the parameter space spanned by *d* and *p*. This fact indicates that an excessive amount of Ca^2+^ in the cytosol, due to small intercluster distances or low values of pump strength, prevents an orchestrated calcium release needed for the wave initiation and propagation. On the other hand, for high values of *d* and *p*, the initial trigger signal decreases before inducing the CICR mechanism, diminishing the probability of wave formation. In the region between these two extreme conditions, where global events are observed, we found a frontier between two regions: one where propagating waves are more abundant than abortive waves, and the other region where abortive waves prevail (Fig. 5). We found that short distances between release sites and small values of pump strength promote the presence of propagating waves. In previous work, Bugrim *et al*. [14] also studied the transition between these two kinds of waves as a function of a parameter related to the release site distance. In such work, the abortive waves are a consequence of a local poor density of release sites, due to a random spatial distribution of deterministic channels, rather than to the intrinsically stochastic nature of the opening/closing channels, as considered here. Despite this fact, they also found that short distances between the release sites promote the propagating waves.

From our model we found that the velocities of calcium waves are in the range 6.6 *μ*m/s < *v_w_* < 9.0 *μ*m/s, in agreement with some experimental results in fertilizing eggs [36]. Also, we saw that the velocity of wave propagation decreases with *d* and *p* (Fig. 8), according to [10, 11, 14]. Our results indicate that the communication between clusters, a crucial ingredient in order to generate waves by the CICR mechanism, is less efficient for high intercluster distance or pump strength. Different theoretical studies have also found that the velocity of calcium waves drops significantly as the distance between release site increases [10, 14]. However, there is experimental evidence from *Xenopus* oocytes supporting that the velocity of calcium waves increases with the pump strength [6]. Falcke *et al*. [9] argue that the experimental findings can be correctly reproduced when the increased SERCA density entails a higher Ca^2+^ content in the ER. According to these authors [9] raising only the SERCA density should generate a decrease in velocity. In our model, the efflux rate does not affect the concentration of luminal calcium, consequently we do not expect to reproduce the experimental results of Camacho *et al*. [6]. However, recent work about the role of luminal Ca^2+^ in the intracellular Ca^2+^ oscillations points out that the fast recovery of Ca^2+^ in the ER lumen seems to be due to Ca^2+^ buffers rather than to the effect of Ca^2+^ pumps [37, 38].

Keizer *et al*. [12] have studied the velocity of calcium waves as a function of the Ca^2+^ coefficient diffusion in different deterministic models. For large intercluster distances they found that the speed of calcium waves is proportional to the diffusion constant, whereas for small distances it is proportional to $\sqrt{D}$. The former mode of propagation is associated with isolated Ca^2+^ release, whereas the latter is related to many sites that simultaneously release Ca^2+^. In the present work, we assumed that calcium wave velocity is proportional to $\sqrt{D}$, since there are many sites releasing Ca^2+^ simultaneously. Therefore, we are able to apply Luther’s law to the propagating calcium waves obtained from our model. Luther proposed a phenomenological equation that establish a relationship between the velocity of a traveling wave and both the diffusion coefficient of the propagator species and an apparent first-order rate constant for the autocatalytic production of the propagator [34, 35]. In fact, Luther’s law has given excellent order-of-magnitude predictions of experimental mitotic wave speeds [39, 40], and also in calcium wave fronts [13, 36]. Here, we used Luther’s law to estimate an effective first-order rate constant of the cytoplasmic calcium release. By adjusting a linear model to the squared velocity of wave front derived from our simulations, we found that the behavior of the effective rate constant for autocatalytic calcium production is consistent with a linear decrease with both the intercluster distance and the pump strength.

Summing up, we believe that the use of hybrid models, which take into account stochastic effects due to the random opening and closing of calcium channels, can offer new insights into the nature of calcium signaling, especially in the context where the hierarchy of different size events is the focus of the modeling.
